# Radiation Risk in Cold War Mexico: Local and Global Networks

**DOI:** 10.1007/s00048-022-00331-0

**Published:** 2022-05-10

**Authors:** Ana Barahona

**Affiliations:** Department of Evolutionary Biology, School of Sciences, Universidad Nacional Autónoma de México (UNAM), Mexico City, Mexico

**Keywords:** Radiobiology, radiation protection, IAEA, safety standards, Cold War Mexico, Laguna Verde

## Abstract

After WWII, global concerns about the uses of nuclear energy and radiation sources in agriculture, medicine, and industry brought about calls for radiation protection. At the beginning of the 1960s radiation protection involved the identification and measurement of all sources of radiation to which a population was exposed, and the evaluation and assessment of populations in terms of the biological hazard their exposure posed. Mexico was not an exception to this international trend. This paper goes back to the origins of the first studies on the effects of radiation and on radioprotective compounds in the Genetics and Radiobiology Program of the National Commission of Nuclear Energy founded in 1960, at a time when the effects of radiation on living beings and radiation protection demanded the attention of highly localized groups of scientists and the creation of international as well as national institutions, and its connection to dosimetry and radiation protection until the 1990s. This historical reconstruction examines the circulation of knowledge, scientists, and their material and cognitive resources, to show that radiobiology, with dosimetry and radiation protection as cases in point, not only were carried out with high international standards in parallel with international agencies, but also reflected local material needs, including the standardization of new experimental techniques.

## Introduction

In December 2013, a truck carrying medical equipment with a source of Cobalt 60 was stolen from a gas station in the state of Hidalgo on the border of the State of Mexico. This equipment, which had been used for radiotherapy, belonged to the Mexican Social Security Institute (Instituto Mexicano del Seguro Social, IMSS), and was being decommissioned. The truck, which bore the words “Transportes Ortiz,” on its side, had left the northern border city of Tijuana on November 28. It was heading toward a radioactive waste repository in the State of Mexico, but the driver stopped to rest at a gas station in the community of Tepojaco in Hidalgo, just 19 kilometers away from its destination. The driver said he was sleeping inside the truck when two men with guns approached the vehicle at 1:30 a.m. on Monday. These criminals tied his hands and feet and abandoned him in a nearby vacant lot. When the driver broke free, he ran back to the gas station for help (La Redacción, Azteca Noticias 2013; Revista Proceso 2013).

While criminals disposed of the radioactive material about 40 kilometers far from the gas station in an ejido of the municipality of Hueypoxtla,^1^ surrounded by 200 hectares of corn field, an alert was issued in seven states to find the stolen truck. Finally, local peasants who were making their daily rounds found the pieces and dragged them to their home; it was then that the municipal authorities informed the police that they had found the radioactive material. Federal authorities immediately went to the area, set up a provisional barrier and gave notice to the National Institute for Nuclear Research (Instituto Nacional de Investigaciones Nucleares, ININ), the National Commission of Nuclear Safety and Safeguards (Comisión Nacional de Seguridad Nuclear y Salvaguardas), and the International Atomic Energy Agency (IAEA) to take up the case.^2^ They also called for the criminals to surrender because of the danger of the stolen material.

This story appeared in all national newspapers, and the Mexican authorities and the IAEA believed that citizens were safe and will remain safe. The UN nuclear agency—the news also said—was to remain in close contact with Mexican authorities to advise them of the protocol to be followed in the event of an accident of this type. The agency acknowledged that the actions taken so far in response to the discovery of the radioactive source were appropriate and followed the protocols for such events (Revista Proceso 2013). Unit 262 of the Department of Radiological Protection (Departamento de Protección Radiológica, DPR) of the ININ traveled to the restricted zone to remove the device, and a group from the Biology Department of the ININ in charge of the biological dosimetry project performed the protocol studies, consisting of cytogenetic analyses of dicentric chromosome on a family of the field workers from Hueypoxtla, State of Mexico, who had found the stolen device, and a group of individuals presumed guilty of stealing the truck. It was possible to establish the dosage values for each of them (close to 28 mSv). In the end, both the family and the thieves were discharged from the hospital where they had been admitted and held for a week. Nowadays, the ININ is the only one in the country with the capacity and personnel to deal with this kind of emergency.

After WWII, global concerns on the uses of nuclear energy and radiation sources in agriculture, medicine, and industry, called for greater guidance in radiation protection. At the beginning of the 1960s radiation protection involved the identification and measurement of all sources of radiation exposure of the population, and the evaluation and assessment of the latter in terms of the biological hazard to the exposed groups. The evaluation included identifying the potential sources of radiation exposure, which ranged from abnormally high natural radiation levels in certain regions, to a nuclear accident of major proportions. These concerns led to the growth of many international research programs for measuring or detecting radiation, controlling exposure to radiation, and exploring the effects of irradiation (Creager & Santesmases 2006; Lindee 2005; Creager 2013). Mexico was not an exception of this international trend.

The story I present in this article goes back to the origins of the first studies on the effects of radiation and on radioprotective compounds, at the Genetics and Radiobiology Program (Programa de Genética y Radiobiología, PGR) of the National Commission of Nuclear Energy (Comisión Nacional de Energía Nuclear, CNEN).^3^ The program was founded in 1960 by British-educated Mexican physician Alfonso León de Garay, at a time when the effects of radiation on living beings and radiation protection demanded the attention of highly localized groups of scientists and the creation of international as well as national institutions. The work done at the PGR provides an important lens through which we can study the postwar intellectual and institutional development of radiobiology (following Rader 2006).

Some questions arise: what was the role that de Garay played in the development of radiation protection programs at the PGR? Who ran the programs and what experimental practices did they use? Were these programs supported by the IAEA? Were there discrepancies between the national concerns and the international agenda on radiation protection? This article aims to look closer at the history of radiation protection in Cold War Mexico and to place it in the context of international concerns about the effects of ionizing radiation on living populations. I will do so by focusing on the PGR’s different research projects from the 1960s to the 1990s supported by the CNEN and the IAEA, when the study of radiation protection agents became international and national priorities. Mexico’s engagement with these issues in ways that reflected the local conditions and international opportunities sheds light on the varied consequences of the global study of radiation risk in this period. In the PGR, the systems established to assess radiation risk focused on protective compounds against radiation effects in the flatworm *Planaria* and on field studies using the fly *Drosophila* before the construction of the nuclear plant in the state of Veracruz.

I will show that the development of radiobiology (including radiation protection) cannot be understood without looking at the development of nuclear physics and the creation of the CNEN in Mexico, and the role played by de Garay in setting up the PGR. Although his participation was tangential to the projects on radiation protection, his participation as a scientist-diplomat-politician was crucial for stabilizing the connection with the IAEA. Also, I attempt to take a more sustained look at the interplay between the PGR, the CNEN, and the IAEA, which fostered the circulation of ideas and scientific practices beyond transnational borders, to see if what happened in Mexico reflected both the global networks of resources and training and the unique circumstances in Mexican institutions and networks.^4^

My narrative will begin with a brief description of the creation of the CNEN and the founding of the PGR. I then turn to address the radiation protection projects at the PGR and then at the Genetics and Radiobiology Department (Departamento de Genética y Radiobiología, DGR). Finally, the article concludes with an examination of the *Drosophila* projects at the Biology Department (Departamento de Biología, DB) in the 1990s, when radiation protection programs were pursued with the support of the IAEA.

## Nuclear Physics in México During the Cold War and the CNEN

After the Mexican Revolution in 1910, a period of political instability lasting until the 1920s set in. At this point, the institutionalization of the Revolution began and was consolidated with the creation of the Institutional Revolutionary Party (PRI, Partido Revolucionario Institucional) in 1929. New political actors took advantage of this instability to obtain better status and privileges. The post-revolutionary governments created new institutions to consolidate the social welfare project for the modernization of the Mexican state. Although this is not the place to delve into the history of collaboration between Mexico and the US, it is worth mentioning that during the post-revolutionary period, many connections were established between Mexican researchers and the leading academic centers in countries such as the US and Great Britain. These ties provided the foundations for further international projects in the post-1945 framework of university and government cooperation. However, at the national level, the academic context was framed by the rise and consolidation of the Mexican State at the national level. It is in this context that nuclear physics and genetics and radiobiology were intertwined in Mexico, where the agendas and interests of prominent Mexican scientists were aligned with national political interests, in accordance with international concerns.

After 1945, international interest in understanding the effects of radiation on human beings led to the formation of institutions and a proliferation of projects, one of which was the founding of the IAEA. On December 8, 1953, at the 470th Plenary Meeting of the United Nations General Assembly, US President Dwight D. Eisenhower addressed the “Atoms for Peace” initiative and recommended the creation of an international organization to regulate the process for the creation and use of atomic energy. He proposed that countries with nuclear technology projects contribute economical and technical resources to the development of peaceful uses of nuclear energy.^5^ In 1957 the IAEA was created with its headquarters in Vienna. The member countries of the UN were invited to participate by creating their own national institutions, which could then attend relevant meetings and benefit from the knowledge produced and the control mechanisms at the IAEA. Several of these organizations were created before, or concurrently with, the formalization of IAEA in 1957: this was the case for CNEN. The creation of IAEA increased funding sources for nuclear scientists and technicians with an emphasis on the peaceful uses of radioisotopes in medical genetics and agriculture.^6^ The CNEN was created in 1956 and highlighted the efficiency of nuclear energy as an energy source, as it was considered a more economical energy source than oil, coal or hydropower. Since its inception, the Mexican nuclear program has had civilian and not military purposes, so the word “atomic” was excluded because of its association with war and the arms race. Instead, the word “nuclear” was included, due to its peaceful connotations (Vélez Ocón 1997; Barahona 2009; 2015a).

The primary fields of interest at CNEN were energy and non-energy applications of nuclear power, as well as nuclear science studies, with nine programs that included nuclear physics, education and training, seminars, reactors, radioisotopes, industrial risks from radiation, agronomy, genetics, and radiological protection. Land and resources for the institute were obtained thanks to the efforts of US-educated Mexican physicists Manuel Sandoval Vallarta, Nabor Carrillo and Carlos Graef, who initiated the study of nuclear physics in Mexico. In 1964, the construction of the Nabor Carrillo Nuclear Center in the town of Salazar, State of Mexico, began, and both a Triga Mark III reactor designed and constructed by General Atomic in the US and a Tandem Van de Graaff positive ion accelerator from the General Electric Company in the US were acquired with the support of the IAEA. These facilities and equipment were intended to foster research on the peaceful uses of nuclear energy and training specialized personnel. Additionally, they were to give support to the National University of Mexico (Universidad Nacional Autónoma de México, UNAM) and the National Polytechnic Institute (Instituto Politécnico Nacional, IPN) (Barahona 2009).

CNEN connected research in nuclear physics and genetics and radiobiology in Mexico with the foundation of the PGR. As Luis Campos has shown, the intersection of the physical and the biological spheres emerged at the turn of the twentieth century, but changed drastically over the second half, giving rise to various new experimental systems and approaches (Campos 2015). In the Mexican scenario, this intersection of fields initiated the opening of new spaces for the development and research of genetics and radiobiology. These spaces included the education and training of the personnel of the PGR in highly specialized international institutions, the collaboration and cooperation with internationally renowned scientists who eventually went to Mexico to give advice and training, and the support of international and national agencies and institutions such as the IAEA and the CNEN.^7^

## The Genetics and Radiobiology Program (PGR) and the Pioneering Work of Alfonso León De Garay

After WWII, the Galton Laboratory at the University College in London was widely considered to be the cradle of human genetics, and Alfonso León de Garay’s (1920–2002) two-year stint there allowed him to learn the most up-to-date techniques that were in the process of being developed, standardized, and used for the measurement of the effects of radiation on human populations and the environment. Upon his return from the Galton Laboratory, de Garay founded the first PGR in Mexico within the CNEN, under the auspices of the IAEA in 1960.

While in London, de Garay became acquainted with the international network of researchers in human genetics. These contacts also facilitated the assimilation and modification of genetic practices in Mexico in the early 1960s. The decade witnessed global trends in human genetics that reshaped the field of biomedicine in the aftermath of WWII, corresponding to growing international interest in understanding the effects of radiation on human beings. Such widespread interest led to the formation of institutions and a proliferation of multi-centered clinical trials and inter-laboratory studies across the globe (Cambrosio et al. 2006a; 2006b; 2009). Mexico was no exception to this trend. After decades of development in plant genetics, investigations of agricultural, animal, and particularly human genetics were also being institutionalized: at the Mexican Institute of Social Security (Instituto Mexicano del Seguro Social, IMSS) headed by Spanish-born and British-educated Mexican physician Salvador Armendares, and at the Hospital for Nutritional Diseases (Hospital de Enfermedades de la Nutrición, HEN), headed by the US-educated Mexican physician Rubén Lisker. The former aimed to study congenital malformations in newborns using cytogenetic techniques, while the latter pursued the study of genetics in indigenous populations using genetic markers (see Barahona 2009; 2015b; 2016).

De Garay studied medicine in the 1940s at the Autonomous University of Puebla, Mexico, and worked for many years as a neurologist.^8^ In 1957, he decided to study population genetics with British medical geneticist Lionel Penrose, the Head of the Galton Laboratory, an authority on human genetics and mental retardation (see Harris 1973; Bewley 2000; Ramsden 2013). Thanks to an agreement between the IAEA and several European universities, de Garay obtained a grant and went to London. The relationships he established in Europe were extremely influential on the PGR he founded upon his return to Mexico in 1960 and had a profound and lasting importance in his later career as a scientist-diplomat-politician (Barahona 2009; 2015a).

As an attendee at the 1957 General Assembly of the IAEA as a companion to the English representatives, he met the Mexican delegation composed of the President of the CNEN, José María Ortiz Tirado, Nabor Carrillo and Manuel Sandoval Vallarta, and the Secretary General of the CNEN, Salvador Carmona (Barahona 2009; 2015a). As a result of this meeting, and with the support of the Mexican delegation, de Garay went on to study the mutagenic effect of radiation, in line with projects that were being developed in other parts of the world. In 1957, with the support of both Ortiz Tirado and Alexander Hollaender, head of the Division of Biology at the Oak Ridge National Laboratory of the US Atomic Energy Commission, de Garay was able to visit Oak Ridge and become well-grounded in the field of radiobiology (Villalobos-Pietrini et al. 2005; Barahona 2015a). His return to Mexico was hastened because the CNEN insisted on the immediate set-up of a laboratory, as a result of rising international pressure on Mexico to develop its own research in genetics and radiobiology. While attentive to the international concerns about the genetic effects of nuclear radiation, CNEN officials assumed that their membership to the IAEA permitted them to develop programs and local laboratories on genetics and radiobiology that could contribute to global knowledge. In light of this opportunity, de Garay accepted the offer to return.^9^

Once in Mexico and while serving as director of the program, de Garay continued to expand his background in the field. He continued to attend international trainings (including those held through the Weizmann Institute in Israel and at Johns Hopkins Hospital in the US) and systematically built a network of intellectual and political exchange. From 1969–70, he served as a member of the group of experts at the UN that was charged with examining the effects of the acquisition, proliferation, possession, and development of nuclear weapons. He also served as an advisor to the Mexican Delegation at the meetings of the UN Scientific Committee on the Effects of Radiation (SCEAR) and was designated Expert *ad personam* by the UN General Secretary U Thant. From 1971–72, he was the Alternate Representative to the IAEA and the UN Organization for Industrial Development (Villalobos-Pietrini et al. 2005).

The PGR was established in 1960 to develop genetics and radiobiology projects and entered into the international network of human biology programs (see de Garay 1962). It consisted at first of a small staff composed of six young researchers who obtained their bachelor’s degrees in biology in 1960, the director Alfonso León de Garay, and Rodolfo Félix Estrada, chief of the *Drosophila* section. The PGR also included a technician, a secretary, and a service assistant (de Garay 1977; Barahona et al. 2003). There were six sections: the *Drosophila *laboratory (where conventional experimentation as well as computing of mutations, including irradiated stocks took place), cytology and genetic analysis, microphotography and autoradiography, biochemistry and radiochemistry, education, and statistics and social work (population genetics and family studies).^10^

The three main lines of research conducted in the first years after its foundation that were in line with international projects while also responding to the national context were, first, cytogenetic studies of certain abnormalities; second, the study of population genetics in *Drosophila *and in Mexican indigenous groups; and third the study of the effects of radiation on hereditary material (Barahona et al. 2003; Barahona 2009, 2015a). Although all of them were crucial for developing and contributing to genetics and radiobiology in Mexico, I will focus on the last one as it provides a key instance of the interplay between international and national scientific and political agendas regarding radiation protection.

## The Study of the Effects of Radiation on Hereditary Material

Owing to Hermann J. Muller’s studies, it had been known since 1927 that exposure of germ cells to radiation produces diverse genetic alterations (Muller 1927). Thanks to studies with *Drosophila*, which not only turned mutagenesis into a field of study, but also facilitated the use of the vinegar fly as an experimental animal for developing methods of detection, the effect of different sources of radiation on genes and chromosomes was recognized.^11^ Since then, many organisms have been used to research and measure the primary genetic effect of radiation on living beings, particularly on genes and chromosomes (Campos 2015). As an example of the international concerns with this problem, many scientific institutions and programs were created, such as the WHO Expert Committee on Radiation, and the United Nations Scientific Committee on the Effects of Atomic Radiation (SCEAR). In 1955, the UN General Assembly formed the SCEAR with the aim of reviewing the effects of levels of radiation from all sources to which man was exposed.^12^ Although these institutions and the IAEA had strong relationships during the Cold War period, it is worth noting that they had similar and sometimes overlapping research programs and committees led by distinguished scientists from all over the world, that not only reflect the geopolitical equilibrium of postwar international radiation protection programs, but also the internationalization of biomedical research for public health (on the WHO see de Chadarevian 2013; Brown et al. 2006; for biomedicine see Creager 2006; Creager & Santesmases 2006).

In September 1962 the WHO Expert Committee on Radiation met in Geneva. Dr. M G. Candau, Director General, opened the meeting and called for attention to the increasing uses and applications of ionizing radiation in industry, research, and medicine, urging that, “public health authorities [need to] be not only fully aware of the hazards to health associated with ionizing radiation and of their responsibilities in this respect, but also fully prepared to meet these responsibilities” (WHO Technical Report Series 1963: 3). Radiation protection was among these effects. Moreover, the radiation-induced genetic effects had, according to the document, added a new dimension to public health thinking.

For its part, in the 1962 Annual Report, SCEAR stated that “the tremendous headway in the last decade in the analysis of genetic function and genetic material has led to a clearer view of the need for a fuller understanding of the mechanism of radiation mutagenesis,” particularly with respect to genes and chromosomes. The report also acknowledged that, although, “much work has been accomplished, no complete answer to the mechanism of radiogenetics has been given” (SCEAR 1962: 59). This was the international context that pointed to the urgent need to tackle the problem worldwide.

In the early days of the PGR, research on the effects of radiation on hereditary material was introduced as a result of international concerns about the harmful effects of radiation due to fallout and weapon testing, and also to strengthen the Mexican nuclear program. From then on, new opportunities gradually appeared for the peaceful application of nuclear energy and general research within the PGR.

Very early on, and with the support of the IAEA, the PGR received a three-month visit in 1962 from Czech biologist and physician Dr. Hans Kalmus, whom de Garay had met during his time in London. Kalmus was at the time a Technical Assistance Expert of the Agency. In his report on Mexican project IAEA TA MEX‑7 dated in 1962, Kalmus stated that all the sub-departments of the PGR were working accordingly, and said that “information and material were exchanged, visits initiated and other contacts made among others with the following institutions: the newly formed Human Genetics Department of EURATOM in Pavia, Italy; the Galton Laboratory, University College, London; the Oak Ridge Laboratory, USA; the Radioisotopes Section of Harwell, England; among others” (IAEA TA/Mex‑7 1962: 6). Other recommendations to the government of Mexico were “that foreign training as provided by fellowships and similar means has greatly contributed to the solid establishment of the PGR in the short period of 18 months; and that further foreign training of its team and some visits by foreign experts are necessary for keeping the planned expansion on an even keel” (IAEA TA Report 69 1962: 7).

In this same report, Kalmus acknowledged his contact with the director and personnel of the National Indigenous Institute (Instituto Nacional Indigenista), which in conjunction planned a set of expeditions

to two Indian populations, the Chamulas in the San Cristóbal region and the Mixtecas in Jamiltepec. These expeditions were organized by the director of the PGR … which lasted two weeks, contact was made with members of the National Malaria Commission (Comisión Nacional contra el Paludismo) who were working in the same neighborhood: collaboration was also agreed and to some extent practically started with various hematological departments and with a research team investigating the goitrous population. (IAEA TA Report 69 1962: 1)

Although space here does not allow for a deeper exploration of the study of human indigenous populations, it is worth noting that very early in the life of the PGR, international experts were involved in the mapping of some genetic markers such as G6PD variants and its relation to some diseases like favism (Barahona 2009; 2016; 2017). Although Kalmus and de Garay’s team were working with classical genetic markers such as PTC tasting, ear wax and color blindness (Kalmus et al. 1964), a couple of years later, de Garay collaborated with US researchers who used more up-to-date genetic markers such as G6PD variants (Bowman et al. 1966; Barahona 2009).

Kalmus was also critical of some of the work being performed at the PGR, including the part-time appointments of the personnel and the lack of training courses, which created major difficulties for the recruitment of suitable staff. He voiced other critics, for example, during his stay, “bacterial contamination, presumably caused by the over-crowded conditions, impeded work for several weeks; but the situation was handled expertly so that conditions are now back to normal. The expert was encouraged to see the introduction of several useful innovations in culturing and staining techniques” (IAEA TA Report 69 1962: 3). One of the main suggestions of this report was the use of *Drosophila* for population studies with the view of providing models for human populations: “The cytogenetical work of the *Drosophila* laboratory is concerned with salivary gland chromosomes and the study of mitosis both in *Drosophila* and some plant material and it seems sufficiently advanced to permit irradiation studies in the near future” (IAEA TA Report 69 1962: 4). As we will see below, this happened as expected. Kalmus’ assessment proved indispensable in ensuring the continuity of the IAEA and the CNEN funding for ongoing and future projects, as well as for the training of experts abroad.

## Radiation Protection

In the 1960s, one of the most important lines of research at CNEN focused on protective compounds that worked against the effects of radiation and the genetic mechanisms of radioprotective substances at the molecular level. This pursuit represented a first-time national initiative. By the late 1970s, this line of inquiry had resulted in independent laboratories on Dosimetry and Radiation Protection, as the CNEN transformed and was renamed the ININ in 1979. According to the Mexican authorities, the advances in the peaceful uses of nuclear energy, particularly in the production of electricity and its applications in medicine, agriculture, and industry, meant that the program needed to increase its level of scientific activity and concentrate on scientific as well as technical and legal efforts intended to prevent any damage that could arise from nuclear development. For that purpose, the new model 200TL source of X‑rays was acquired, among other equipment, to perform a wide range of thermoluminescence dosimetry. Of great importance were the investigation of genetic damage as an indicator of radioactive environmental contamination and quantitative estimation of the risk that this could lead to a rise in mutation rates and the population’s genetic load. All these techniques and procedures were in accordance with international standards established by the IAEA (Rentetzi 2021; Ito & Rentetzi 2021; see also endnote 1).

In order to be competitive on a worldwide scale, the PGR introduced the Aquatic Invertebrate Radiobiology Section and the Molecular Genetics Laboratory in 1965. The idea was to work, respectively, on protective compounds against radiation effects in the flatworm *Planaria*, and to investigate the genetic mechanisms operating in microorganisms, as well as the radiation effects and consequences of radioprotective compounds at the molecular level. The Project “Radiation protective drugs in aquatic organisms” was supported by the CNEN, the UNAM and the IAEA, and lasted from 1966–1971. The head of the section was young biologist Alfredo Laguarda who had been hired in 1962 and was working at the time in his PhD thesis on the “Effect of X rays, gamma and UV radiation on *Dugesia* (Platyhelmintes: turbularia) under the action of the serotonin-creatinine sulfate complex (S-CS),” under de Garay’s supervision.^13^

A large number of compounds with diverse chemical structures had been found to have radioprotective properties, but “it was known at the time that the S‑CS have been used in numerous experiments as a radioprotective agent. The effectiveness of this substance was considered by Langendorff and colleagues since 1959 as the best radiation protector known at the time. Since then, several hypotheses have been proposed to explain its mechanism. Many authors postulated that S‑CS produced tissue hypoxia because of vasoconstriction, and that it is the decrease in the oxygen tension in the tissues that causes the diminution of damage induced by radiation” (Laguarda & Villalobos-Pietrini 1967: 667). So, it was very important to corroborate this hypothesis using an organism lacking a circulatory system such as *Dugesia*.

The animals were collected from a natural pond on the grounds of the Botanical Garden of the UNAM and they were maintained in the lab in dishes with water and fed with fresh liver. The experimental animals were placed for one hour in the S‑CS solution prior irradiation. Dosimetry was determined by a Siemens Universal Dosimeter. Different groups were irradiated with total exposures of 650 R to 2000 R (X-rays, gamma, and UV rays). The control group was irradiated without any S‑CS treatment. The S‑CS at a concentration of 3.14 × 10^−5^ M, protected the *planaria Dugesia* from the lethal action of radiation. Vasoconstriction and the subsequent lowering of oxygen tension inside the tissues was not involved in this observed radioprotection. Because these experiments were very successful, de Garay asked Laguarda to repeat them with other organisms. Laguarda and other colleagues from the UNAM choose the plant *Vicia faba*, the results being very similar. These experiments were very important because much work had been done using S‑CS in mammals. They reported the results on the radioprotection of *Planaria* and *Vicia faba* by the same complex (serotonin-creatinine sulfate complex, S‑CS), expanding the knowledge of radioprotective substances on living beings (Villalobos-Pietrini & Laguarda 1968).

Very soon after these experiments on radioprotection compounds were being developed, IAEA radiogenetics expert Gregorio Olivieri traveled to Mexico in April 1967 for a three-month visit. In his report he stated that,

the laboratory on aquatic invertebrates with various species of *Planaria*, are successfully raised. Using characteristics of the circulatory system of these organisms, studies on the mechanism of action of serotonin, a radioprotective agent, are being performed. Cytogenetics studies are also being performed on other organisms referring to *Vicia faba*. (IAEA Report 335 n.d.: 5)

Additionally, he planned to carry out several experiments with the new *Drosophila* stocks, to look for genetic damage in larvae feed with radioisotopes. He hoped to work in cooperation with the tissue culture groups, but this proved impossible since de Garay was not in Mexico at the time to give his authorization.

As part of the staff training, annual courses about radioisotopes and nuclear instrumentation were organized in the Radioisotope Laboratory at the Physics Department of the School of Sciences of the UNAM in close collaboration with the CNEN. These courses were compulsory for the personnel who worked in the Program.^14^ During Laguarda’s years at the PGR he attended the radioisotopes and instrumentation courses given by de Garay and Mexican chemist Augusto Moreno y Moreno, whom he had met at the IAEA meeting in Vienna in 1957 (Barahona 2009). When Laguarda left the PGR in 1970 to work as a full-time professor at the UNAM, the project on aquatic organisms was abandoned and the lab was closed, but the idea to use radioprotective substances on other organisms was followed by other young researchers at the PGR particularly in *Drosophila*. The interest of pursuing these studies in the program not only focused on determining the damage caused by radiation, but also worked to test some agents to evaluate the protective or inhibitory action of some substances with *Drosophila* flies, specifically the chlorophyllin that had already been cataloged as a radioprotective compound.

## Radiation Protection at the Genetics and Radiobiology Department in the 1980s

Years later in 1972, after CNEN became the National Nuclear Energy Institute (Instituto Nacional de Energía Nuclear, INEN),^15^ and moved to the Nabor Carrillo Nuclear Center in Salazar, State of Mexico, the PGR was reorganized, and renamed Genetics and Radiobiology Department (Departamento de Genética y Radiobiología, DGR). María Esther de la Rosa, formerly student of de Garay, who had joined the CNEN in 1964 as a technician of the *Drosophila* laboratory, became the head of the department and, along with friend and colleague Matilde Breña, embarked on a study to measure the effects of radiation in natural populations. In the late 1980s de la Rosa supervised the MD thesis of biology student Citlali Guerrero on dosimetry and radioprotection, and years later, in 1994, she hired her in the department. In 1996 and 1997 Guerrero went to England to study under the supervision of Dr. David Lloyd at the National Radiation Protection Board, now the Center for Radiation, Chemical and Environmental Hazards of Public Health England. Since the 1990s, Guerrero was (and still is, as of writing) in charge of the Biological Dosimetry Laboratory in close collaboration with the IAEA. Guerrero’s team is responsible for performing cytogenetic analysis to measure the effects of radiation on human populations.^16^

In the 1970s, there were negotiations with the federal government to construct a nuclear electric station in Laguna Verde, Veracruz, by the Federal Electricity Commission (Comisión Federal de Electricidad, CFE) with the technical advice of the INEN. In 1972 the CFE signed a contract with General Electric for the purchase of the reactor, and with Mitsubishi for the turbogenerator. After several years of delay due to problems with the tenders and with the Single Union of Electrical Workers of the Mexican Republic (SUTERM), which opposed its construction, finally, in October 1976, the construction of Unit 1 started, only to begin commercial operations in 1990. In the case of Unit 2, its construction began in 1977 and it joined the power grid in 1995. The construction of Laguna Verde was part of the Mexican nuclear energy program initiated in the 1960s. It constituted an attempt to evaluate the capacity of the research and development systems to carry out a state-of-the-art technological project. According to some authors, Laguna Verde was a laboratory for the country’s modernization since this project included negotiations between the union (the SUTERM) and the CFE, the political projects for Mexico to enter electrical self-sufficiency, the Mexican scientific and technical capacity, and the collaboration of international institutions such as the IAEA (Azuela & Talancón 1999).^17^ The construction of the nuclear plant mobilized not only political and financial resources (local and international) and international cooperation programs, but also scientific and technical ones, among which were the initiatives to measure radiation in natural populations of the INEN, particularly in the group of the de la Rosa, Matilde Breña and their students, noting the friction between the local and global agendas.

Since the 1960s, it was widely known that “the possibility of local or generalized airborne contamination of the environment with radioactive substances arises in connection with a wide range of atomic energy activities, including the operation of various types of nuclear reactors” (WHO Technical Report Series 1963: 20). Years later, in September 1972, Mexico hosted the sixteenth regular session of the IAEA General Conference, held for the first time in a Latin American country. The IAEA wrote, “in view of the role that Mexico had played in arms control and disarmament in Geneva, New York and Tlatelolco, it was most appropriate for the Agency to hold its second General Conference away from Headquarters in Mexico City” (IAEA General Conference 1972: 2). One of the main objectives of the conference was to approve the cooperation agreement between the AIEA and the Organization for the Prohibition of Nuclear Weapons in Latin America (OPANAL), set up under the Tlatelolco Treaty. The conference considered some expanded activities, among which were the assessment of the environmental impact of nuclear power programs, the study of appropriate environmental monitoring around nuclear installations, and estimation of population dose exposure from radioactive contaminants present in the environment.

In the Mexican report given at the conference, the then President of the INEN, physicist Fernando Alba Andrade noted that

preliminary studies for the building of a nuclear power plant had started five years previously. However, the stage had now been reached at which the CFE, after examining tenders submitted by companies in various countries, had placed an order for the reactor for the first unit of the power station at Laguna Verde, Veracruz … having received valuable assistance from the Agency, which had sent two missions to advise on the most important aspect of the project. (IAEA General Conference 1972: 5)

The report also said that the Mexican government would soon submit to the Agency a request for technical assistance in preparing draft regulations for construction and safe operations, and in obtaining the fuel for the power station. As INEN authorities and attendees at the conference, Manuel Sandoval Vallarta, Carlos Graef, and Salvador Cardona, among others, provided support to the Mexican scientists at the DGR to conduct a series of experiments using *Drosophila* as a target population prior to the construction of the Laguna Verde Nuclear Plant. For these purposes, personnel of the DGR initiated the project IAEA MEX/7/007 “Molecular genetic analysis of the effects of Low-level radiation” in 1972, to be approved by the IAEA Technical Cooperation and Assistance Department. This project aimed to study the radiation effects on sibling *Drosophila* species from Laguna Verde. This was a long-term study of species *D*. *melanogaster* and *D. simulans* located in Laguna Verde.^18^

To identify and quantify the possible effects of background radiation due to the generation of electricity in nuclear electric plants, the behavior of natural populations of *Drosophila* living in the vicinity was analyzed over a ten-year period. In a long-term study to measure the radioprotective action of some chemical compounds, Breña and de la Rosa embarked in a survey of *Drosophila* populations using monosodium glutamate (MSG), ascorbic acid (vitamin C), chlorophyllin, and other radioprotective compounds (for the study of the material and epistemological culture of Drosophila genetics, see Velasco 2019). The objective was to indicate the biological effects, if any, of the operation of the nuclear reactor in surrounding populations. This project attracted the attention of other groups working on the effects of radiation and radioprotective compounds, and collaboration soon began with scientists from the Program in Biology, Brown University, and the Department of Biology, City College of New York. Grants were given to conduct parts of this project, once again demonstrating the international collaborative networks that had been established in the wake of WWII.^19^

The fly larvae were fed using micropipettes with chlorophyllin, then irradiated with a gamma irradiator with Cobalt 60 for one minute. The fly adults were put in alcohol and using the SMART technique^20^ their eyes were analyzed looking for spots as evidence of genetic mutations. In the case of the wings, the procedure was the same: if mutations occurred the hairs were deformed, or there were 3 hairs per cell (normally flies have one hair per cell). If the mutation frequency was low, they could assure that chlorophyllin acted as radioprotective compound (Pimentel et al. 1996; Zimmering et al. 1992; 1990; Aguilera et al. 1993; Olvera et al. 1993a; 1995). The antecedent of this project was “The study of radiological impacts through biological indicators” run by de la Rosa in the early 1980s. Some results of this project were presented at several international meetings, including the First International Conference on Biological Aspects of the Consequences of the Chernobyl Power Station Accident in the USSR in 1990, at which de la Rosa was part of the organization panel of experts, and at the Chernobyl Ecological Network in 1992 (Levine et al. 1992). In the years to come, de la Rosa and colleagues embarked on the organization of several international conferences at the ININ and the UNAM, recognizing that Mexican scientists were experts in the field of radiobiology and instrumental parts of international collaborative networks.

Beyond investigating biological effects of the operation of the nuclear reactor in surrounding populations, another line of research of this project was to study other physiological and somatic characteristics in *Drosophila* populations to assess changes due to radiation exposure. Among these were species abundance, viability, desiccation resistance, and dispersal. These studies were conducted in close collaboration with international drosophilist networks, showing that the studies performed were critical, as they contributed to the general knowledge on the effects of radiation in living organisms (s Guzmán et al. 1989; Levine et al. 1989; and Olvera et al. 1993b).

The last project that de Garay lobbied the IAEA for took place in the 1990s. Its technical proposal, which Matilde Breña presented, included the setup of a molecular biology laboratory to identify the effects of radiation exposure. The efforts made by ININ officials were led by the then head of the DB, de la Rosa. Both women were former students of the program, and professor de Garay was the diplomatic liaison with the IAEA.^21^ Other participants included physicist Javier Reyes from the Environmental Sciences and Genetics Group of the ININ, and Rafael Villalobos-Pietrini from the Center of Atmospheric Sciences of the UNAM. The latter had worked with de Garay’s team back in the 70s with *Vicia faba*.

The proposal intended to set up a molecular biology laboratory at the ININ to identify the effects of radiation in natural populations. The IAEA sent two expert missions to evaluate the project’s feasibility led by David C. Lloyd and Simon Boulfler. Lloyd approved the proposal on his visit in 1993 but considered it very ambitious and suggested it should focus on two main lines: the development of biological dosimetry by classical cytogenetics to identify chromosomal aberrations caused by ionizing radiation, and the use of molecular biological testing. The first involved specific molecular techniques, chiefly using the comet assay,^22^ and the second considered using a molecular biology test through FISH.^23^ Three grants were awarded in the same proposal to Citlali Guerrero, Emilio Pimentel, and Jorge Serment. As head of the project, de la Rosa went to the UK under Lloyd’s supervision to learn lymphocyte culture for chromosomal aberrations assays and sister chromatid exchange and micronucleous techniques. While in the UK, de la Rosa also visited James Perry at the University of Swansea to learn HPRT locus somatical mutational assays. Guerrero and Pimentel went to Harwell to study molecular biology under Lloyd’s supervision. Serment attended Sussex University in the UK to work with Michael Green on molecular cytogenetics and molecular analytical techniques.

One year later, in 1994, the ININ received a visit from the Direction of the Radiation Biological Division of the Life Sciences Ramendra Mukherjee from the IAEA to review the project and, where applicable, refocus the activities and planning requests for experts, equipment and grants. The meeting included physician Javier Reyes, head of the Environmental Sciences and Genetics Group, and Matilde Breña from the DB, both at the ININ, and de la Rosa, who had moved to the Department of Inorganic Chemistry at the UNAM. The report stated that

TA MEX/7/007 holds a recognizable timely importance in its help facilitating public understanding for the acceptance of nuclear technology in making inroads to the national economic advancement and wellbeing. Besides, there are other prospects of the project’s outcome in contributing to any improved understanding of the human health effects of low doses and low-dose-rate radiations including other issues, such as cancer prevention and radiation protection. (IAEA TA/MEX/9/007 n.d.: 5)

## Conclusions

In the aftermath of World War II, international and local institutions were formed not only to provide technical assistance (like the IAEA and the CNEN), but also to foster public health programs around the world (like the WHO and the PGR). After 1945, nuclear science witnessed the increasing presence of scientists in diplomatic affairs and the need for international cooperation as a precondition for scientific collaboration. At the same time, the IAEA’s global role in regulating and solving nuclear problems was strengthened (see Ito & Rentetzi 2021). In this article, we have seen how the technical cooperation between the CNEN and the IAEA, although asymmetric in certain scientific and technical aspects, was conducted collaboratively and in adherence to regulatory nuclear policies. Following Ito and Rentetzi (2021), the political processes and knowledge production in nuclear science happened together. As with the IAEA and many other international and national agencies, the CNEN (including the PGR) also came to be a scientific and political institution that dominated nuclear research and radiobiology in the national setting, as it cooperated with international organizations in the global circulation and control of scientific knowledge. As Maria Rentetzi has written “the precondition for any scientific collaboration among nations has been their political cooperation” (Rentetzi 2017: 40).

Radiobiology began to be studied in Mexico at the PGR founded by de Garay in 1960 upon his return from England as a result of international concerns about the effects of nuclear radiation on living beings. My historical reconstruction paid attention to the circulation of knowledge, scientists, and material and cognitive resources, to show that radiobiology, with dosimetry and radiation protection as cases in point, were not only carried out with high international standards in parallel with international agencies, but also reflected local material needs, including the standardization of new experimental techniques. I also attempted to show that radiation protection and the role that the IAEA had in Mexico’s genetics and radiobiology developmental research programs are spaces for historical inquiry that allow us to follow the itineraries and trajectories of knowledge production (“knowledge on the move”), as well as to observe the effects of radiation on living populations, a characteristic of the transnational perspective in the history of science (Safier 2010; Secord 2004; Turchetti et al. 2012).

Looking back at the founding of the PGR and its contribution to the field, its work cannot be understood without paying attention to the development of nuclear physics and the creation of the CNEN and its development under the auspices of the IAEA, and to the international movement towards the pacific uses of nuclear energy. I attempted to show that de Garay’s role as a scientist-diplomat-politician played a key role in the construction of institutions and interventions across national borders. Thanks to his membership in an international network dedicated to human genetics, as well as to the Mexican scientific elite of the 1960s, de Garay was seen as both an authority at the local level and the international one. This dual authority allowed him to act as a scientific liaison between the CNEN and the IAEA, to obtain the necessary support, technical and economical, for the completion of the research projects and the training of the staff of the PGR, as part of his personal agenda.^24^ As he was not involved directly in many of the projects that were being developed at the PGR (he did not co-author most of the published results), by the 1980s he had left the scientific planning activities and the administrative tasks of the program, and given more responsibilities to other scientists. The PGR engagement and experience in radiobiology and radiation protection received support from both the CNEN and the IAEA to conduct several lines of research, to incorporate young researchers, and to send many of them off to attend international centers and major universities to acquire the most advanced techniques and knowledge in the areas of genetics and radiobiology. The interplay between the PGR, the CNEN (later INEN and ININ), and the IAEA from the 1960s to the 90s fostered the circulation of ideas and practices beyond national borders. Also, the scientific and technological cooperation with the IAEA in the name of promoting international peace and security as part of the expansion of programs for technical assistance, fostered research on radiation protection in the country (Miller 2006; Fischer 1997).

Among the first studies on radiation protection in Mexico were the ones performed by Laguarda on radioprotective compounds that were not well known at the time. Although this line of research ended when Laguarda left the PGR, others would later continue it with *Drosophila* years later. Of particular interest, was the work done by de la Rosa and her Mexican and international colleagues, in the achievement of contributing to the global knowledge on radiation protection. The work done by this international research network, in which de la Rosa and others participated, to establish the first nuclear plant in Laguna Verde, was very important since it opened spaces for new research lines and experimental techniques, and also enabled the training abroad of Mexican young scientists. De La Rosa’s participation in the conference in Chernobyl and part of the Chernobyl Ecological Network, along with various publications on the matter, allowed her and her colleagues to be regarded internationally as scientific experts whose authority derived from their belonging to international networks.

Nowadays, the objective of biological dosimetry and radiation protection networks is to pool human and technical resources in the event of a radiological emergency, as well as to conduct intercalibration exercises for different assays. Putting the contributions of the PGR, the DGR, and the DB to radiation protection into perspective, the Department of Radiological Protection (DPR), which is now part of the Radiological Safety Management of the ININ, aims to monitor the Institute’s radiological safety conditions and provide internal and external services. It has two laboratories: Environmental Radiological Surveillance and the Internal Dosimetry Laboratory. To carry out its objective, the DPR also supports essential services provided in other areas, such as the calibration of measuring equipment, radioactive waste disposal and medical service, among others. Also, the ININ now has the only Biological Dosimetry Laboratory in Mexico. Both departments have been able to attend to radiological emergency situations. One of these was the robbery that took place in 2013, when a truck was stolen containing medical equipment that used a source of Cobalt 60. Guerrero and her team performed the chromosome analysis (using dicentric chromosomes) to estimate the exposure dose to the family of the peasants who initially were in contact with the radioactive source in the corn field, and to the group of thieves who were already in custody; Unit 262 of the DPR then removed the container and took it to the radioactive waste repository.

## Acknowledgements

I would like to thank MC Alicia Villela for her expert research assistance and the two reviewers whose suggestions substantially improved the manuscript. María Esther de la Rosa, Citlali Guerrero, and Alfredo Laguarda deserve special thanks for helping me with this research. Their interviews, suggestions, and the documents they made available to me improved this manuscript. I would like to thank my friends and colleagues with whom, over the last 27 years, I have been sharing my ideas when I pioneered the study of the history of genetics and radiobiology in Cold War Mexico in the context of the international peaceful uses of nuclear energy after I returned to Mexico in 1995 after having spent a one-year postdoctoral stay with Francisco J. Ayala at UC Irvine. Among them are Luz Fernanda Azuela, Marcos Cueto, Mike Dietrich, Judith Guzmán, Hans-Jörg Rheinberger, Susana Pinar, Marsha Richmond, María Jesús Santesmases, and Ana Romero de Pablos. This manuscript has benefited from these collaborative networks. I also want to thank Maria Rentenzi and Martin Kusch for inviting me to the “Claiming Authority, Producing Standards” meeting held at the University of Vienna in June 2016. Finally, I want to thank the IAEA in Vienna for helping me with the archival research during my stay in June 2016, especially Archive Director Leopold Kammerhofer and Martha Riess for helping me at a distance and in situ. This research was partially financed by CONACyT A1-S-8786, and PAPIIT/UNAM IN403718.

## Footnotes

1 Ejido or communal land, constitutes the economic sustenance of the community life of the family nuclei.

2 The IAEA has specific safety standards, that serve as a global reference for protecting people and the environment. They were launched in 1958 but are under periodical review since then. Although safety is a national responsibility, Member States (like Mexico), have adopted the IAEA standards for national regulations. Also, there are many international conventions relating to nuclear and radiation related activities that Member States have subscribed. Among them are the Convention on Early Notification of a Nuclear Accident, and the Convention on Assistance in the Case of a Nuclear Accident or Radiological Emergency. These conventions place obligations to the countries where some of these accidents might occur (see IAEA Safety Standards n.d.; Fischer 1997, chapters 7 and 8).

3 The CNEN became the National Institute for Nuclear Energy (Instituto Nacional de Energía Nuclear, INEN) in 1972, and ININ in 1979.

4 If we think of international science as “the” international science, as somehow all the same, we are missing the richness of the special qualities and adaptations of scientific knowledge and practices when crossing epistemic and geographical borders. I would like to thank one of the reviewers for bringing this point to my attention.

5 As Krige has pointed out, the Atoms for Peace initiative was a polyvalent policy intended to divert attention from the military to the benign atom (Krige 2006).

6 Before WWII biology, agriculture, and medicine served to represent the peaceful face of atomic energy, but it was only after WWII that medical practices and biological knowledge changed drastically (see Creager & Santesmases 2006).

7 As Miller has pointed out, international scientific and technological cooperation programs changed drastically after WWII, when they were used as “effective instruments of peace” (Miller 2006) (for cooperation and collaboration see also Beatty 1993).

8 De Garay, personal communication with author, August 1998.

9 It was also attractive since his doctoral studies were becoming increasingly complex, and there were few guarantees of continuing with a scholarship. De Garay, personal communication with author, August 1998.

10 At its peak in the 1970s and 1980s, the PGR included about 30 researchers, ten technicians and many undergraduate students both from the UNAM and the IPN, two of the major educational institutions in Mexico.

11 In the late 1950s there were disputes about the proper way to apply results with *Drosophila* to human radiation risk. One of these disputes has been documented by Jacob Hamblin between American geneticists Alfred H. Sturtevand and Hermann J. Muller, with the US National Academy of Science’s 1956 study on the Biological Effects of Atomic Radiation (Hamblin 2007). Lindee has documented the controversies and discussions between, Muller and American human geneticist James V. Neel, regarding the genetic effects of radiation in a “context of a ‘New World’ of nuclear risk” (Lindee 2013: 207).

12 It is worth noting that de Garay and Rodolfo Félix, both working at the PGR, were appointed in 1972 as scientific experts and members of national delegations (SCEAR Report 1972). Another institution formed in the inter-war period was the International Commission on Radiological Protection established in 1928 in the United Kingdom as an independent organization linked to the International Congresses of Radiology. Although the commission started working prior to WWII, it began to publish a series of reports soon after the war in 1958 until the present.

13 Laguarda, personal communication with author, March 2016.

14 Olga Olvera & Judith Guzmán, personal communication with author, September 2001; Laguarda, personal communication with author, March 2016.

15 The INEN became the ININ in 1979, and the DGR became first, the Radiobiology Department (Departamento de Radiobiología, DR), and then the Biology Department (Departamento de Biología, DB).

16 De la Rosa, personal communication with author, April 2016; Guerrero, personal communication with author, May 2016.

17 Although this is not the place to deep into the creation of the Laguna Verde nuclear plant, it is worth saying that it was during the term of President Gustavo Diaz Ordaz (1964–1970) that the nuclear project began given the consolidation of the scientific research with the establishment of the Nuclear Center in Salazar (the INEN including the PGR founded by de Garay), and the Federal Commission of Electricity (CFE), dependent on the federal government.

18 Before constructing the nuclear plant, other studies were planned to measure the hydrologic, climate, and ecological conditions. The Institute of Marine Sciences and Limnology of the UNAM dealt with the ecological and marine structure. At the same time, the School of Biological Sciences of the National Polytechnic Institute (IPN) studied vegetation and soils.

19 The grants were: US Department of Health and Human Services No. 2‑S07-RR07132, and City University of New York PSC-CUNNY Research Award Program to Louis Levine (see de la Rosa et al. 1989a, b; and Rockwell et al. 1991).

20 **S**omatic **M**utation **A**nd **R**ecombination **T**est, with observable markers for wings and eyes.

21 In 1979, the then Mexican President José López Portillo appointed de Garay ambassador to Israel where he stayed until 1983. Upon his return to Mexico, he rejoined the ININ as scientific advisor to director Carlos Vélez, who invited him to develop the project “The genetic effects of the Chernobyl accident,” and as a first step he was invited to Belarus by Nikolai A. Kartel, from the Academy of Sciences of Minsk. In 1994, upon leaving Vélez, de Garay was relieved of his duties.

22 Induced breaks in the DNA molecule of leukocytes (white blood cells) make the molecule to stretch giving the impression of seeing a comet. De la Rosa, personal communication with author, April 2016.

23 This test introduces synthetic DNA probes fluorescently labeled to leukocytes. With a fluorescence microscope, chromosomes can be seen by looking for an alteration or not.

24 As Ortega y Gasset once said, one is one and his circumstance (Ortega y Gasset 1921). In this sense, de Garay, having been born in a wealthy family, and having been part of the scientific elite of the Mexican “miracle,” with highly administrative and diplomatic skills to navigate between the scientific community and the federal authorities, used his means and federal funding to achieve not only a scientific agenda, but also to establish public relations. He was known for his eccentric and lavish receptions to his guests. This practice is very widespread nowadays: the use of federal funding to welcome and host lunches and dinners in fancy restaurants with margaritas, in order to establish social bonds and strengthen international cooperation.
